# ATR-FTIR spectroscopic markers indicating drug resistance in selected *Candida* strains

**DOI:** 10.1038/s41598-025-01428-x

**Published:** 2025-05-24

**Authors:** Agnieszka Dróżdż, Dominika Kubera, Alina Olender, Wojciech Dabrowski, Magdalena Szukala, Sylwia Wosko, Joanna Chwiej, Marzena Rugiel, Kamil Kawoń, Mariusz Gagoś

**Affiliations:** 1 Faculty of Physics and Applied Computer Science, AGH University of Krakow, al. A. Mickiewicza 30, 30-059 Krakow, Poland; 2 Department of Cell Biology, Maria Curie-Sklodowska University, Akademicka 19, 20-033 Lublin, Poland; 3 Department of Medical Microbiology, Medical University of Lublin, Chodźki 1 Street, 20-093 Lublin, Poland; 4 First Department of Anaesthesiology and Intensive Therapy, Medical University of Lublin, Jaczewskiego street 8, 20-090 Lublin, Poland; 5 Laboratory of Preclinical Testing, Department of Applied and Social Pharmacy, Faculty of Pharmacy, Medical University of Lublin, 20-293 Lublin, Poland

**Keywords:** *Candida*, ATR-FTIR spectroscopy, Drug resistance, Multidrug resistance, Antifungal resistance, Spectroscopic markers, Fungi, Infectious-disease diagnostics, Pathogens, Microbiology, Molecular biology, Biomarkers

## Abstract

The rising incidence of fungal infections and the increasing prevalence of antifungal resistance highlight the need for rapid and reliable diagnostic methods. This study investigates the potential of ATR-FTIR spectroscopy to identify spectroscopic markers of drug resistance in selected *Candida* strains. In this pilot study, ATR-FTIR spectroscopy was employed to analyse the biochemical composition of *Candida albicans*, *Candida glabrata* and *Candida dubliniensis* isolates. The minimum inhibitory concentrations (MIC) of antifungals were determined using antifungals concentration gradient strips, and the spectral data were processed to identify differences between resistant and sensitive isolates. Based on the results for *Candida albicans*, *Candida glabrata* and *Candida dubliniensis*, specific ATR-FITR spectroscopic markers of drug resistance were identified, which highlighted the necessity for these markers to be antifungal-specific. Despite the limitations of the study, the findings underscore the potential of ATR-FTIR spectroscopy in identifying spectroscopic markers of antifungal resistance. These preliminary results provide a foundation for further research, which could lead to the development of rapid diagnostic tools for detecting drug-resistant *Candida* strains, thereby improving the management and treatment of fungal infections.

## Introduction

In recent years, there has been an increase in the frequency of fungal infections. Invasive fungal infections are a global problem causing approximately 1.7 million deaths annually^1^. Candidiasis is among the most common fungal infections. Fungi of the genus *Candida* can be part of the natural human microbiota present on the skin and in the digestive and reproductive systems. However, there are many factors that can lead to their excessive growth and infection. Individuals particularly at risk include post-transplant patients, those taking immunosuppressive drugs, subjects undergoing broad-spectrum antibacterial antifungal therapy, the elderly, or hospitalized and immunocompromised patients. Despite their common presence in the microbiota, *Candida* fungi are considered one of the leading causes of hospital-acquired infections^2^.

Among the many strains isolated from clinical samples, *Candida albicans* is most frequently identified (37%), followed by *Candida glabrata* (27%). As reported by Kainz et al., ‘Other clinically relevant species recovered from blood stream infections include *Candida parapsilosis* (14%), *Candida krusei* (2%), *Candida tropicalis* (8%), *Candida dubliniensis* (2%) and *Candida lusitaniae* (2%)’^2^. What is more, multidrug resistant *Candida* isolates have been increasingly identified^3^. Recently, candidiasis has become a major threat to hospitalized patients due to the severe course of COVID-19^4^. The most commonly used drugs to combat fungal infections include antifungals belonging to azoles (fluconazole, voriconazole, posaconazole, itraconazole), echinocandins (anidulafungin, micafungin) and polyenes (amphotericin B). Currently, the determination of pathogen susceptibility to antifungals requires performing a classical antibiogram, which is time-consuming and delays the start of treatment^5^. Therefore, development of a method for rapid and reagent-free determination of pathogen resistance to commonly used antifungals is extremely important from the standpoint of diagnosing and treating fungal infections caused by *Candida* species.

One of the research techniques commonly used in biomedical studies is Fourier transform infrared (FTIR) spectroscopy^6–10^. It provides valuable information about the chemical composition and molecular structure of a wide range of samples by measuring the absorption of infrared radiation (IR). The infrared spectrum contains peaks corresponding to different vibrational modes of molecules in the sample, allowing identification and quantification of functional groups and chemical bonds. FTIR spectroscopy offers several advantages, including high sensitivity, rapid data acquisition and the ability to analyse samples in various states (solid, liquid, gas)^11,12^. Therefore, the method has found applications in diverse fields, such as environmental monitoring^13^, forensic analysis^14^, pharmaceutical quality control^15^ and biomedical research^16^. Currently, FTIR spectroscopy is being developed as a tool to support the diagnosis and/or detection of markers for a range of diseases^17–19^.

In this pilot study, ATR-FTIR spectroscopy was employed to search for markers of drug resistance and multidrug resistance in selected *Candida* strains. This technique allowed us to analyse the molecular composition of the samples, providing detailed insights into the biochemical changes associated with resistance. Identification of specific spectral markers is the first step in the development of a rapid and reliable method for detecting drug-resistant *Candida*, which is crucial for improving treatment strategies and patient outcomes. What is more, the spectroscopic analysis presented in this paper facilitates the identification of phenotypic expression features of genetic resistance mechanisms in the form of changes in the structure of lipids, proteins and cell wall components that are correlated with resistance mechanisms. Genetic methods, often used in studies of resistance mechanisms, detect changes in the genome (DNA) of *Candida* species, such as the presence of mutations^20,21^. However, the key aspect is the phenotypic expression of these genetic changes and its level in the process of the actually functioning resistance mechanism^22–24^. Such analysis is crucial, and the method presented in the paper is highly valuable.

For our studies, we selected *Candida albicans*, *Candida glabrata* and *Candida dubliniensis* strains. *Candida albicans* is the most common cause of candidiasis and a significant contributor to hospital-acquired infections^25,26^. *Candida glabrata* is known for its increasing resistance to azole antifungals, thus posing a growing challenge in clinical settings^27,28^. *Candida dubliniensis*, although less prevalent, shares many characteristics with *Candida albicans* but can exhibit distinct antifungal resistance patterns^29^. By including these strains, we aimed to cover a broad spectrum of clinical relevance and resistance profiles, ensuring comprehensive and impactful results in our search for ATR-FTIR spectroscopic markers of drug resistance.

## Results

The selection of isolates for comparison was based on the minimal inhibitory concentrations (MIC) of antifungals^30^. Clinical breakpoints of MIC established by the European Committee on Antimicrobial Susceptibility Testing (EUCAST) were used to distinguish between antifungals-sensitive (S) and antifungals-resistant isolates (R)^30,31^. For each strain and each antifungals, the spectra of resistant isolates were compared to those of sensitive isolates. ATR-FTIR absorption bands with intensities that differed significantly between resistant and sensitive isolates, as determined by the Mann-Whitney U test, were identified as potential spectral markers of drug resistance. For isolates exhibiting resistance to multiple antifungals, markers of multidrug resistance were determined using the same approach. The occurrence of these markers was analysed only for antifungals for which EUCAST breakpoints have been established^30,31^.

### Antibiograms

The antibiograms of the analysed *Candida* isolates are presented in Tables 1, 2 and 3. These tables include clinical breakpoints for isolates that are sensitive (S) and resistant (R) to specific antifungals. For *Candida albicans*, breakpoints were established for itraconazole (ITC), posaconazole (POS), voriconazole (VO), fluconazole (FLU), amphotericin B (AMB), anidulafungin (AND) and micafungin (MYC). *Candida glabrata* has breakpoints defined for fluconazole (FLU), amphotericin B (AMB), anidulafungin (AND) and micafungin (MYC). For *Candida dubliniensis*, breakpoints are known for itraconazole (ITC), posaconazole (POS), voriconazole (VO), fluconazole (FLU) and amphotericin B (AMB).

Analysis of Table 1 reveals that there are no *Candida albicans* isolates resistant to amphotericin B among the collected samples. Consequently, it is not possible to determine spectroscopic markers of drug resistance for this antifungal drug; hence, it was excluded from further analysis. Moreover, three of the isolates exhibited multidrug resistance to fluconazole, posaconazole, voriconazole and itraconazole (FLU + POS + VO + ITC), while four cases showed simultaneous resistance to anidulafungin and micafungin (AND + MYC). These cases were included in the analysis of spectroscopic markers of multidrug resistance to azoles and echinocandins.

**Table 1:** EUCAST breakpoints and MIC values of *Candida albicans* isolates.

EUCAST MIC breakpoints (mg/L)^31^							
Antifungal	ITC	POS	VO	FLU	AMB	AND	MYC
S ≤	0.06	0.06	0.06	2	1	0.03	0.016
R >	0.06	0.06	0.25	4	1	0.03	0.016

MIC of *Candida albicans* isolates (mg/L)							
Isolate	ITC	POS	VO	FLU	AMB	AND	MYC
0	0.06	0.047	0.064	0.125	0.064	0.008	0.003
36	32	32	32	256	0.75	0.006	0.016
37	32	32	32	256	1	0.006	0.006
40	0.125	32	0.006	0.5	0.125	0.003	0.006
41	0.125	0.012	0.012	0.5	0.125	0.002	0.006
43	0.006	0.006	0.012	0.6	0.19	0.004	0.008
45	32	32	32	256	0.19	0.004	0.012
48	0.25	0.006	0.006	0.25	0.19	0.012	0.008
52	0.25	0.5	0.006	0.25	0.25	0.032	0.012
53	0.125	0.0064	0.006	0.5	0.19	0.032	0.012
54	0.38	32	32	0.25	0.19	0.012	0.008
59	0.19	0.006	0.006	0.5	0.094	0.008	0.008
61	0.125	0.047	0.006	0.19	0.125	0.004	0.004
64	0.006	0.0094	0.016	0.5	0.19	0.012	0.004
65	0.125	0.19	0.25	0.5	0.25	0.003	0.008
69	0.125	0.023	0.008	0.38	0.19	0.016	0.008
71	0.25	0.006	0.012	0.5	0.38	0.003	0.006
72	0.75	0.19	0.094	0.75	0.25	0.032	0.008
74	0.25	0.064	0.006	0.25	0.125	0.012	0.006
75	0.125	0.047	0.006	0.5	0.19	0.008	0.006
84	0.125	0.19	0.008	0.5	0.125	0.12	0.006
87	0.125	0.0012	0.125	0.25	0.094	0.016	-
88	0.75	0.19	0.032	0.75	0.064	0.016	0.004
89	0.25	32	0.25	0.5	0.25	0.023	0.006
95	0.25	0.012	0.25	0.5	0.25	0.047	0.004
96	0.125	0.25	0.25	0.5	0.19	0.023	0.012
97	0.75	0.38	0.064	0.5	0.38	0.002	0.006
101	0.125	0.12	0.25	0.5	0.5	0.016	0.006
102	0.125	0.047	0.125	0.5	0.25	0.008	0.004
104	0.75	0.002	0.25	0.5	0.25	0.032	0.0016
105	0.38	0.006	0.012	0.25	0.25	0.047	0.0016
106	0.125	32	0.25	0.5	0.25	0.032	0.012
107	3	32	0.094	0.5	0.19	0.016	0.008
110	0.75	0.25	0.064	0.75	0.19	0.004	0.008
111	0.25	0.19	0.023	0.5	0.125	0.004	0.006
112	0.75	0.25	0.047	1	0.25	0.008	0.003
113	0.125	32	0.023	0.5	0.19	0.008	0.003
114	0.125	32	0.023	0.5	0.25	0.012	0.004
116	0.75	0.19	0.047	0.75	0.064	0.006	0.004
117	0.5	0.19	0.032	1	0.19	0.016	0.008
121	0.125	0.094	0.008	0.5	0.064	0.008	0.003
123	0.25	0.125	0.125	0.5	0.003	0.094	0.003
124	0.094	0.064	0.008	0.38	0.094	0.016	0.004
125	0.125	0.032	0.125	0.5	0.19	0.012	0.008
126	0.094	0.016	0.006	0.25	0.125	0.004	0.002
127	0.125	6	0.19	12	0.125	0.012	0.006
129	0.125	0.064	0.006	0.25	0.19	0.008	0.004
131	0.094	0.064	0.023	1	0.25	0.032	0.023
132	0.125	0.064	0.023	1	0.19	0.032	0.023
133	0.012	0.023	0.023	0.5	0.19	0.016	0.023
134	0.125	0.064	0.064	0.5	0.125	0.064	0.032
135	0.094	0.047	0.047	0.5	0.25	0.047	0.023
136	0.094	0.004	0.094	0.38	0.38	0.002	0.047

Based on the MIC values listed in Table 2 and the EUCAST breakpoints, it can be observed that there are no *Candida glabrata* isolates sensitive to amphotericin B and only one isolate shows resistance to micafungin. Consequently, these antifungals were excluded from further analysis.

**Table 2:** EUCAST breakpoints and MIC values of *Candida glabrata* isolates.

EUCAST MIC breakpoints (mg/L)^31^				
Antifungal	FLU	AMB	AND	MYC
S ≤	0.001	1	0.06	0.03
R >	16	1	0.06	0.03

MIC of *Candida Glabrata* isolates (mg/L)				
Isolate	FLU	AMB	AND	MYC
3	2	0.38	0.094	0.006
14	24	0.75	0.094	0.008
25	24	0.5	0.125	0.006
28	6	0.38	0.064	0.006
47	0.5	0.25	0.064	0.006
60	8	0.25	0.125	0.016
80	1	0.38	0.047	0.012
100	2	0.38	0.064	0.008
102	16	0.38	0.032	0.008
118	12	0.25	0.006	0.006
120	0.5	0.38	0.094	0.064
122	0.75	0.25	0.032	0.006
1–84	2	0.19	0.006	0.008

As shown in Table 3, none of the collected *Candida dubliniensis* isolates exhibit resistance to fluconazole and amphotericin B. Therefore, similar to the other strains, these antifungals were excluded from further analysis.

**Table 3:** EUCAST breakpoints and MIC values of *Candida dubliniensis* isolates.

EUCAST MIC breakpoints (mg/L)^31^					
Antifungal	ITC	POS	VO	FLU	AMB
S ≤	0.06	0.06	0.06	2	1
R >	0.06	0.06	0.25	4	1

MIC of *Candida Dubliniensis* isolates (mg/L)					
Isolate	ITC	POS	VO	FLU	AMB
32	0.125	0.094	0.012	0.250	0.250
42	0.500	0.250	0.023	0.750	0.064
44	0.125	0.064	0.012	0.750	0.125
50	1.000	0.380	0.064	1.500	0.064
55	0.019	0.125	0.032	1.000	0.064
56	0.250	0.125	0.047	1.000	0.064
57	0.380	0.190	0.012	0.190	0.047
76	0.190	0.094	0.016	0.500	0.125
85	0.380	0.125	0.320	0.750	0.094
86	0.064	0.032	0.006	0.750	0.047
93	0.094	0.032	0.016	0.500	0.047
109	0.023	0.016	0.004	0.190	0.047
138	0.125	0.25	0.064	1	0.25
143	0.064	0.032	0.006	0.25	0.047

### Identification of ATR-FTIR absorption bands in spectra of analysed *Candida* species

**Fig. 1:**
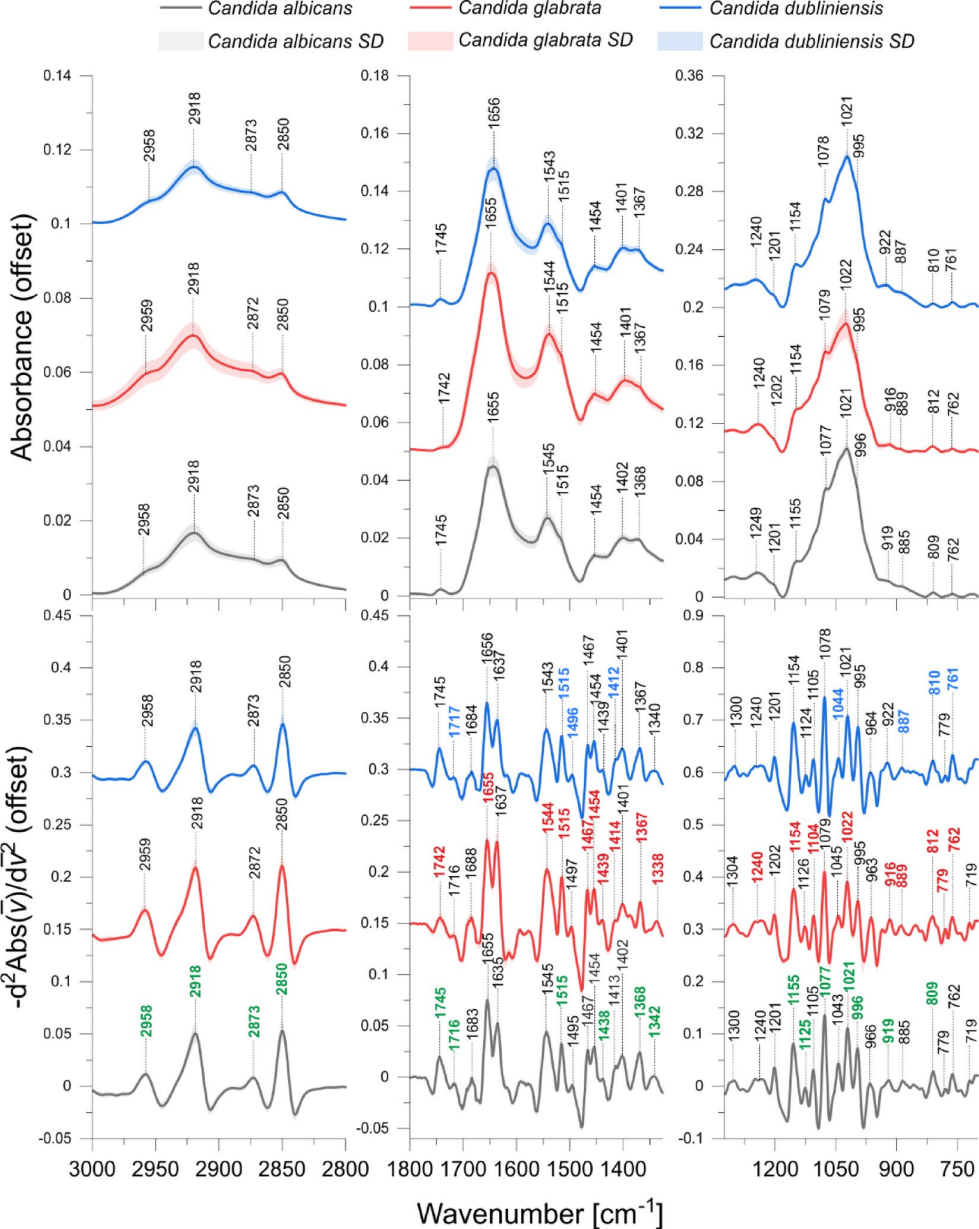
Mean ATR-FTIR spectra of *Candida albicans*, *Candida glabrata* and *Candida dubliniensis* (upper charts) and their corresponding reversed second derivatives (lower charts). Standard deviations (SD) of spectra were marked on charts as light coloured regions. The identified absorption bands are marked in the charts. Additionally, the specific marker bands discussed in the manuscript are marked in green for *Candida albicans*, in red for *Candida glabrata* and in blue for *Candida dubliniensis*.

The mean ATR-FTIR spectra of *Candida albicans*, *Candida glabrata* and *Candida dubliniensis* as well as their reversed second derivatives are presented in Fig. 1. Second derivatives of the spectra help to enhance spectral differences and resolve the issue of overlapping components in IR absorption bands^32^. In the reversed second derivative, the maxima correspond directly to the peaks in the original FTIR spectrum. This simplifies the identification of spectral bands and enhances the clarity of the features, especially in complex spectra. In contrast, in the conventional second derivative, the minima correspond to the maxima in the original spectrum, which can sometimes complicate the interpretation and make it harder to discern the true positions of the peaks. By reversing the second derivative, we make the peaks more directly comparable to those in the original spectrum, improving the accuracy of spectral analysis^32,33^. The integrated area under specific peaks in the reversed second derivative was used as a measure of band intensity. Although the second derivative reflects curvature, the integrated area within a defined peak region in the reversed second derivative still correlates with the abundance of the underlying biochemical components. This approach improves the resolution of overlapping bands and reduces baseline effects, while maintaining a proportional relationship between the integrated signal and the abundance of the corresponding biomolecular components^32–34^. Therefore, the spectral assignments were based on these derivatives, as presented in Table 4.

**Table 4:** Identification of ATR-FTIR bands in *Candida* species^35–50^.

Band (cm^−1^) or band ratio (cm^−1^/cm^−1^)			Assignment*	Origin/characteristics	
*Candida* *albicans*	*Candida* *glabrata*	*Candida dubliniensis*			
2958	2959	2958	v_as_(CH_3_)	Lipids	
2918	2918	2918	v_as_(CH_2_)		
2873	2872	2873	v_s_(CH_3_)		
2850	2850	2850	v_s_(CH_2_)		
1745	1742	1745	v(C=O)	Lipids, phospholipids, esters	
1716	1716	1717			
1683	1688	1684	v(C=O), δ(NH)	AI	Amide I proteins, antiparallel β-sheet and β-turn
1655	1655	1656			Amide I proteins (peak), α-helix
1635	1637	1637			Amide I proteins, β-sheet
1545	1544	1543	δ(NH), v(CN)	AII	Amide II proteins
1515	1515	1515			
1495	1497	1496			
1467	1467	1467	δ(CH_2_), δ(CH_3_)	1360–1480 cm^−1^	Lipids and proteins
1454	1454	1454			
1438	1439	1439			
1413	1414	1412			
1402	1401	1401			
1368	1367	1367			
1342	1338	1340	δ (CH_2_)	Phospholipids and amino acids	
1300	1304	1300	δ(NH)	Amide III proteins	
1240	1240	1240			
1201	1202	1201	v_as_(PO_2_^-^)	Phosphomannan	
1155	1154	1154	C–O–C	β(1 → 3) glucan	
1125	1126	1124	v(CO), v(CC)	α-glucan, RNA	
1105	1104	1105	v(CO), v(CC), v(COC)	Glycogen and β(1 → 3) glucan	
1077	1079	1078	v_s_(PO_2_^-^), v(CO), v(CC), δ(COH)	Glycogen, phosphomannan, DNA	
1043	1045	1044	v(CO), v(OH)	Glycogen, mannan	
1021	1022	1021	v(CO)	Glycogen	
996	995	995	v(CC), δ(CO)	β(1 → 6) glucan	
966	963	964	v(CC), δ(CO)	Mannan	
919	916	922	v(CC), δ(CO)	Glucan, mannan	
885	889	887			
809	812	810	δ (CH)	Glucan	
779	779	779			
762	762	761			
719	719	-			
2918/2958	2918/2959	2918/2958	v_as_(CH_2_)/v_as_(CH_3_)	Changes in lipid chain length, branching and/or saturation level	
2850/2873	2850/2872	2850/2873	v_s_(CH_2_)/v_s_(CH_3_)		
AII/AI	AII/AI	AII/AI	Amide II/Amide I	Changes in protein structure	
1635/1655	1637/1655	1637/1656	β-sheet/α-helix	Changes in secondary structure of proteins	
1125/1077	1126/1079	1124/1078	RNA/DNA	Changes in gene expression	

*Types of vibrations: v_as_—asymmetric stretching, v_s_—symmetric stretching, δ—deforming vibrations.

Figure 1 Mean ATR-FTIR spectra of *Candida albicans*, *Candida glabrata* and *Candida dubliniensis* (upper charts) and their corresponding reversed second derivatives(lower charts). Standard deviations (SD) of spectra were marked on charts as light coloured regions. The identified absorption bands are marked in the charts. Additionally, the specific marker bands discussed in the manuscript are marked in green for *Candida albicans*, in red for *Candida glabrata* and in blue for *Candida dubliniensis.*

The intensities of the absorption bands identified for the analysed *Candida* species were calculated as the integral peak areas based on the reversed second derivatives of the IR spectra. The intensity of a given absorption band is proportional to the abundance of the biomolecule that contains this band. Additionally, ratios of selected absorption bands, as shown in Table 4, were calculated to provide insight into changes in the lipid and protein structure as well as gene expression. The values of these spectral parameters were compared between *Candida* isolates exhibiting resistance and sensitivity to specific antifungals. The statistical significance of the observed differences was evaluated using the Mann-Whitney U test at significance levels of *p* < 0.05 and *p* < 0.1. Absorption bands and band ratios that differed significantly between the resistant and sensitive isolates were identified as potential spectroscopic markers of drug or multidrug resistance. The results of statistical analysis are presented in Tables 5 and 6.

### ATR-FTIR spectroscopic markers of drug resistance and multidrug resistance to selected antifungals

For all analysed *Candida* species, a number of statistically significant differences in the biochemical composition were observed between isolates that were resistant and sensitive to the antifungals selected for the study. These differences show their potential as markers of resistance to these antifungal drugs. Furthermore, some of the analysed spectral parameters may also serve as markers of multidrug resistance to selected azoles and echinocandins.

However, in the context of spectroscopic markers of drug resistance, it is crucial that the identified markers are specific to a given antifungal. This specificity implies that the intensity of the marker absorption band should exhibit significant changes only between isolates that are resistant and those that are sensitive to the particular antifungal in question. It should not vary between isolates that are resistant or sensitive to other antifungals. This characteristic ensures that the marker can reliably indicate resistance to a specific drug without being confounded by resistance to other medications, thus enhancing the precision of diagnostic tests. For example, the effectiveness of a biomarker depends on its specificity, which directly affects its ability to provide accurate diagnostic information and avoid false-positive results in the presence of resistance to other antifungals.

#### *​Candida albicans*

While analysing Table 5, several parameters can be identified that meet the specificity assumptions necessary for markers of drug resistance to fluconazole, posaconazole, micafungin and multidrug resistance to anidulafungin and micafungin in *Candida albicans*. None of the analysed spectroscopic parameters exhibited specific changes that could be indicated as markers of resistance to voriconazole, itraconazole and anidulafungin or as markers of multidrug resistance to azoles.

The fluconazole-resistant isolates exhibited a significant increase in the intensity of the 1716 cm^−1^ band originating from C= O stretching vibrations present in lipids, phospholipids and esters as well as the 1515 cm^−1^ and amide II bands originating from proteins. Additionally, increases were observed in the 1360–1480 cm^−1^ range corresponding to lipids and proteins, the 1125 cm^−1^ band assigned to α-glucans and RNA and the 809 cm^−1^ band originating from glucan, compared to the sensitive isolates. Conversely, a significant decrease in the intensity was noted for the band at 1368 cm^−1^ assigned to lipids and proteins.

Compared to the sensitive isolates, the posaconazole-resistant isolates showed significantly decreased intensities in the 2958 cm^−1^, 2918 cm^−1^ and 2850 cm^−1^ bands originating from lipids, the 1745 cm^−1^ band assigned to lipids, phospholipids and esters as well as a relative intensity decrease in the 2850/2873 cm^−1^ ratio associated with changes in the lipid structure. Statistically significant increases were observed for the 1155 cm^−1^ band associated with β(1→3) glucan, the 1077 cm^−^¹ band originating from glycogen, phosphomannan and DNA, the 1021 cm^−^¹ band of glycogen, the 996 cm^−1^ band corresponding to β(1→6) glucan and the 919 cm^−1^ band of glucan and mannan.

In the case of multidrug resistance to anidulafungin and micafungin, specific decreases in the 2873 cm^−^¹ absorption band of lipids and the 2918 cm^−1^/2958 cm^−1^ relative intensity associated with changes in the lipid structure were present in the resistant isolates, compared to the sensitive ones.

The micafungin-resistant *Candida albicans* isolates, compared to the sensitive cases, exhibited a decrease in the intensity of the 1438 cm^−1^ band, which is associated with lipids and proteins. Conversely, in the resistant isolates, statistically significant increases were observed for the 1342 cm^−1^ band assigned to phospholipids and amino acids, the AII/AI band ratio corresponding to structural changes in proteins and the 1125 cm^−^¹/1077 cm^−^¹ band ratio indicating changes in gene expression.

**Table 5:**
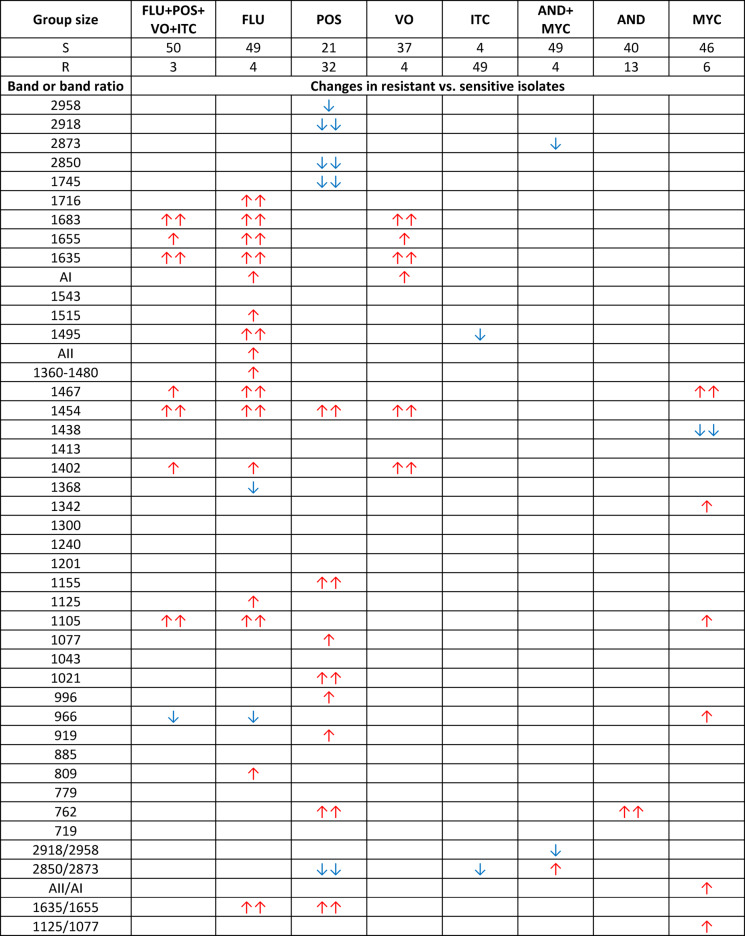
Results of the Mann–Whitney U test for *Candida albicans*.

Statistically significant increases in the intensities of the analysed spectral parameters in isolates resistant to a given antifungal compared to sensitive ones are marked with red upward arrows, while decreases are marked with blue downward arrows. Differences significant at the level of *p* < 0.05 are indicated by double arrows and those significant at the level of *p* < 0.1 are indicated by single arrows. For each antifungal, the number of sensitive (S) and resistant (R) cases is provided.

#### *Candida glabrata*

Based on Table 6, several biochemical differences were observed between the fluconazole- and anidulafungin-resistant *Candida glabrata* and their corresponding sensitive groups. Some of the analysed parameters that showed significant changes between resistant and sensitive isolates can be indicated as specific spectroscopic markers of drug resistance to these antifungals.

*Candida glabrata* exhibiting resistance to fluconazole revealed significant increases in the 1655 cm^−^¹ band of α-helix proteins, the 1104 cm^−^¹ band assigned to glycogen and β(1→3) glucan and the 2850 cm⁻¹/2872 cm⁻¹ band ratio associated with structural changes in lipids. In turn, statistically relevant decreases were observed for the 1467 cm^−^¹ band corresponding to lipids and proteins, the 889 cm^−^¹ band assigned to glucan and mannan as well as the 779 cm^−^¹ and 762 cm^−^¹ bands originating from glucan.

The anidulafungin-resistant isolates, compared to the sensitive ones, presented increased intensity in the 1742 cm^−^¹ band of lipids, phospholipids and esters, the 1544 cm^−^¹ and 1515 cm^−^¹ bands of amide II proteins, the 1454 cm^−^¹, 1439 cm^−^¹, 1414 cm^−^¹ and 1367 cm^−^¹ bands associated with lipids and proteins, the 1338 cm^−^¹ band assigned to phospholipids and amino acids, the 1240 cm^−^¹ band corresponding to amide III proteins, the 1154 cm^−^¹ band of β(1→3) glucan, the 916 cm^−^¹ band of glucan and mannan and the 812 cm^−^¹ band associated with glucan. Conversely, a specific statistically significant decrease in intensity was observed at the 1022 cm^−^¹ band of glycogen in the resistant isolates compared to the sensitive group.

#### *Candida dubliniensis*

*Candida dubliniensis* exhibited severalspecific changes in the biochemical content that may serve as potential spectroscopic markers of drug resistance to posaconazole, voriconazole and itraconazole, as shown in Table 6.

In the posaconazole-resistant isolates, compared to the sensitive cases, there was a statistically significant decrease in the 1412 cm^−1^ absorption band associated with lipids and proteins, the 887 cm^−1^ band related to glucan and mannan and the AII/AI ratio, which indicates changes in the protein structure. Statistically relevant increases were observed for the 1044 cm^−1^ band associated with glycogen and mannan and the 810 cm^−1^ band assigned to glucan.

In the voriconazole-resistant isolates, there was a decrease in the 1717 cm^−1^ absorption band associated with lipids, phospholipids and esters. In contrast, relevant increases were noted for the 1515 cm^−1^ and 1496 cm^−1^ bands corresponding to amide II proteins.

Resistance to itraconazole in the *Candida* isolates was manifested solely by a decrease in the intensity of the 761 cm^−1^ absorption band assigned to glucan.

#### Summary of the results

To facilitate interpretation, the observed spectroscopic changes in resistant versus sensitive *Candida* isolates can be grouped according to the major biomolecular components affected. Changes in lipid content and structure were among the most consistent findings, reflected in altered intensities and band ratios in the CH stretching region (2958, 2918, 2873, 2850 cm) and ester-related bands (1745, 1742 cm⁻¹), which are characteristic of lipids and phospholipids^35,46^. These changes suggest modifications in me^−1^mbrane composition, consistent with known resistance mechanisms involving ergosterol biosynthesis disruption and compensatory lipid remodelling^51,52^. Alterations in protein structure and content were also frequently observed, particularly in the Amide I and II regions (1655, 1635, 1545, 1515 cm⁻¹), as well as in the AII/AI ratio, indicating possible changes in secondary structure or protein expression under stress conditions^35,47^. Furthermore, changes in glucans, mannans, and phosphomannan structures were evident in bands associated with polysaccharides and nucleic acids (1155, 1125, 1077, 1043, 996 cm⁻¹), pointing to modifications in the fungal cell wall — a hallmark of resistance, particularly to echinocandins^38,44,49,53^. The diversity and specificity of these biochemical alterations underscore the utility of ATR-FTIR spectroscopy for profiling antifungal resistance across multiple *Candida* species.

**Table 6:**
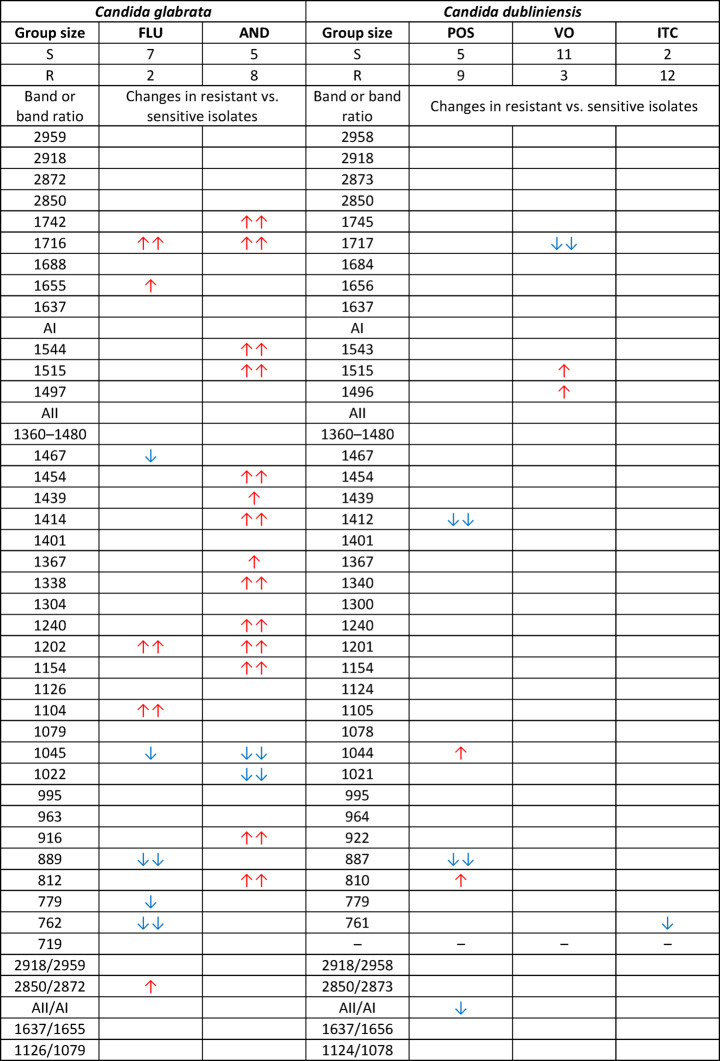
Results of the Mann–Whitney U test for *Candida glabrata* and *Candida dubliniensis.*

Statistically significant increases in the intensities of the analysed spectral parameters in isolates resistant to a given antifungal compared to sensitive cases are marked with red upward arrows, while decreases are marked with blue downward arrows. Differences significant at the level of *p* < 0.05 are indicated by double arrows and those significant at the level of *p* < 0.1 are indicated by single arrows. For each antifungal, the number of sensitive (S) and resistant (R) cases is provided.

## Discussion

In the conducted studies, ATR-FTIR spectroscopy was used to analyse biochemical changes in *Candida* strains resistant to various antifungal drugs belonging to azoles, echinocandin and polyene groups to identify spectroscopic markers of drug resistance. It is crucial that the identified resistance markers are specific to a given antifungal to reliably indicate resistance to a particular antifungal drug without being confounded by resistance to others. The spectroscopic analysis allowed the identification of changes in the structure of lipids, proteins and cell wall components, which may be related to resistance mechanisms.

What is important, fungal resistance in *Candida* species can develop in response to antifungal treatment. Over time, repeated or inappropriate use of antifungal agents can lead to selective pressure, encouraging the survival and proliferation of resistant strains^54^. This resistance can become established within a given strain or isolate if it continues to encounter the same or similar drugs, leading to a more permanent resistance profile. Such resistance is often associated with specific genetic mutations or adaptations that alter the target sites of antifungal drugs or enhance the ability of the fungi to expel the drugs^25,55^. Consequently, the resistant traits can be passed on to subsequent generations, making the resistance a persistent feature of that strain or isolate^54,55^. This highlights the importance of careful antifungal use and ongoing monitoring of resistance patterns in clinical settings.

In *Candida albicans* strains, specific biomolecular changes observed in isolates with resistance to selected antifungal antifungals may be linked to mechanisms underlying this resistance. In the case of isolates resistant to fluconazole, i.e. an azole antifungal which targets ergosterol synthesis, there are notable changes in the intensity of bands associated with lipid, protein and glucan content. This may be the result of metabolic and structural adaptations of fungal cells in response to treatment^51,56,57^. Resistance to antifungal drugs often involves significant modifications in the membrane lipid composition^51,52,58^. The changes in the intensity of lipid-related bands in FTIR spectra suggests that resistant strains may increase the synthesis of alternative lipids or sterols to compensate for the loss of ergosterol^51^. Posaconazole-resistant isolates exhibit alterations in the lipid content and structure as well as changes in glucans, mannans, phosphomannans and glycogen. These changes suggest that resistance mechanisms involve changes in the cell wall architecture, potentially affecting drug penetration or binding^53,54,59–62^. Furthermore, the observed modifications in the lipid structure could influence membrane fluidity and permeability, contributing to the reduced efficacy of posaconazole^51^.

In the case of multidrug resistance to anidulafungin and micafungin, significant changes in the lipid content and membrane structure are noted. Echinocandins inhibit β(1,3)-D-glucan synthesis, a vital component of the fungal cell wall. Resistance to echinocandins is often associated with changes in β(1,3)-D-glucan levels and the overall cell wall composition^63^. Alterations in lipid profiles might affect membrane fluidity and permeability, which can hinder the effectiveness of echinocandins^55,63^. In turn, micafungin-resistant *Candida albicans* solely presented changes in the protein content and structure as well as modifications in the expression of genes that may be related to cell wall synthesis and stress response; this suggests that resistant strains have developed adaptive mechanisms to counteract the effects of echinocandins^64,65^.

The resistance of *Candida glabrata* to fluconazole and anidulafungin can be attributed to several molecular mechanisms that lead to changes in the protein and lipid composition. In the case of fluconazole resistance, mutations in the ERG11 gene, which encodes the target enzyme in the ergosterol synthesis pathway, can reduce drug binding^51^. Additionally, increased expression of efflux pumps can expel the drug from the cell, lowering its intracellular concentration^54,57^. The results obtained suggest changes in lipid structures, likely due to compensatory increases in other lipid components resulting from disruptions in the ergosterol pathway^51,66^. Moreover, the activation of stress responses plays a significant role in resistance mechanisms. These responses often involve the upregulation of heat shock proteins (HSPs) and other chaperones that assist in protein folding and protect against stress conditions induced by antifungal drugs, which may explain the changes in protein levels^64,65^.

In *Candida glabrata*, resistance to azoles, such as fluconazole, can be partly linked to alterations in β-(1,3)-glucan levels. Enhanced β-(1,3)-glucan synthesis might contribute to drug resistance by improving the fungus’s ability to bind and sequester antifungal drugs^62^. Additionally, glycogen is closely associated with β-(1,3)-glucans in the cell wall. Glycogen and β-(1,3)-glucans are covalently linked and contribute to the structural integrity of the cell wall^53,61,67^. This association may play a role in reinforcing the cell wall, thus influencing fungal resistance to antifungal agents. By modifying cell wall components, including glycogen and glucans, *Candida glabrata* can adapt to and survive under antifungal drug pressure. Cell wall remodelling is a significant mechanism of resistance to echinocandins. Echinocandins inhibit β-1,3-glucan synthase, a critical enzyme in cell wall synthesis. Mutations in the FKS1 and FKS2 genes reduce drug binding and efficacy, leading to increased synthesis and remodelling of cell wall components^55,59,63^. The changes observed in the ATR-FTIR spectra of the anidulafungin-resistant *Candida glabrata* suggest modifications in proteins and lipids, likely reflecting increased synthesis or alterations in cell wall components to compensate for the weakened glucan network^55,63,68^.

The changes observed in the FTIR spectra of the posaconazole- and voriconazole-resistant *Candida dubliniensis* isolates highlight various biochemical adaptations that likely contribute to antifungal resistance. The alterations observed in the posaconazole-resistant isolates include changes in the lipid and protein content and in the protein structure. These modifications can potentially affect the permeability and fluidity of the cell membrane, making it more difficult for posaconazole to penetrate and exert its antifungal effects^51^. Additionally, modifications in membrane proteins can have an impact on the drug’s binding sites, reducing its efficacy or altering enzyme functions and structural proteins to adapt to the presence of the drug^56,60^. Changes in the intensities of bands related to glucan and mannan also indicate structural modifications in cell wall polysaccharides. Since glucan and mannan are crucial components of the fungal cell wall, their alteration could reduce drug binding and penetration^54,69^. The increased absorption bands related to glycogen, mannan and glucan suggest enhanced glycogen storage and structural changes in the cell wall. Glycogen accumulation can provide an energy reserve to combat drug-induced stress, while modifications in glucan could contribute to drug resistance by binding and sequestering antifungal drugs^61,62^.

Alterations in lipids, phospholipids and esters were observed in the voriconazole-resistant *Candida dubliniensis*. These changes may indicate shifts in the lipid composition of the cell membrane, potentially decreasing drug uptake and altering membrane integrity^51,57^. Phospholipids are vital for membrane fluidity and function, and their modification could hinder the ability of voriconazole to disrupt the cell membrane^51,56^. Furthermore, changes in amide II bands suggest modifications in enzyme and structural protein content, which might be a response to maintain cellular functions under drug pressure^51,62,64^.

The observation of a decrease in the intensity of the absorption band assigned to glucan in the itraconazole-resistant *Candida dubliniensis* isolates suggests a challenging interpretation in the context of antifungal resistance mechanisms. Typically, increased levels of glucan are associated with enhanced resistance to antifungal agents^62^. A decrease in glucan might initially suggest a reduction in the protective capabilities of the cell wall, which could theoretically lower resistance. Moreover, the decrease observed in this study was statistically significant at a level of 10%. This relatively small magnitude of change combined with the potential for statistical Type I error due to the small sample sizes and/or disproportions in the groups studied raises concerns about the robustness and reliability of the result. The observed decrease in glucan might not necessarily reflect a true biological effect but could instead be an artefact of the limitations of the study. Further research with larger sample sizes and more controlled conditions is needed to clarify the role of glucan in itraconazole resistance and to confirm whether this decrease is a valid indicator of resistance mechanisms or a statistical anomaly.

The presented study has several limitations that could affect the results and their interpretation. One notable limitation is the small number of isolates for some *Candida* strains, with only 13 cases of *Candida glabrata* and 14 cases of *Candida dubliniensis*, due to their limited clinical availability. Additionally, the limited number of isolates with resistance and sensitivity to certain antifungals could affect the robustness of the comparisons. The significant disparities between the numbers of resistant and sensitive isolates may introduce bias and limit the generalizability of the findings. Another limitation is the use of a 10% significance level (*p* < 0.1) in the analysis. This approach is justified in the exploratory phase of the research to detect potential trends and directions for further investigation, especially given the limited sample size and the preliminary nature of the study. While this lower threshold helps identify promising markers that warrant more extensive studies with larger sample sizes in the future, it also increases the risk of false positives.

An important limitation of this pilot study is the lack of advanced statistical classification and validation methods such as Receiver Operating Characteristic (ROC) analysis, which is widely used for evaluating the diagnostic performance of biomarkers^70^. Additionally, chemometric methods such as Principal Component Analysis (PCA), Linear Discriminant Analysis (LDA), and other pattern recognition techniques can significantly enhance spectral resolution, facilitate dimensionality reduction, and reveal subtle differences in complex biological spectra^71–73^. However, the application of chemometric methods requires the use of large data sets due to the high dimensionality and complexity of spectral data, which demand robust statistical power to detect subtle but meaningful patterns. Sufficient sample size also ensures reliable training, validation, and generalization of multivariate models such as PCA or LDA, which are essential for accurate classification and biomarker identification^74^. Although our current study focused primarily on the identification of statistically significant spectral markers using univariate analysis, future work involving larger datasets will benefit from the integration of these multivariate and classification techniques. Their implementation may allow not only for the validation of biomarker performance but also for the development of predictive models to support rapid, spectroscopy-based diagnostics of antifungal resistance.

The use of manual integration intervals based on the extreme positions of peaks in the mean spectrum for each Candida species may pose a limitation when analysing larger datasets. While this approach ensured accuracy in a small pilot dataset, future studies with larger sample sizes will require automated optimization of integration windows to enhance scalability and reproducibility. This step would enable more efficient and objective analysis in larger studies.

These limitations could have an impact on the accuracy and reliability of the identified spectroscopic markers of drug resistance, potentially affecting the overall conclusions of the study. Future research with larger sample sizes and more balanced groups is needed to validate these findings and ensure more comprehensive results.

## Conclusion

Despite the limitations of this study, the research conducted to identify spectroscopic markers of drug resistance is significant. The small number of isolates for some *Candida* species and the limited number of isolates with resistance and sensitivity to certain antifungals present challenges. Additionally, the significant disparities between the numbers of resistant and sensitive isolates could introduce bias and limit the generalizability of the findings. Nevertheless, the findings from this pilot study provide a valuable foundation for future research.

The observed statistically significant specific differences in the biochemical composition of resistant and sensitive *Candida* strains highlight the potential of ATR-FTIR spectroscopy as a diagnostic tool. The identification of specific spectral markers associated with drug resistance can enhance our understanding of the mechanisms underlying antifungal resistance. Furthermore, these markers may eventually lead to the development of rapid and reliable diagnostic methods for detecting drug-resistant *Candida* strains, which is crucial given the rising incidence of fungal infections and the increasing prevalence of antifungal resistance.

Current diagnostic methods for fungal infections are often time-consuming and may delay the initiation of appropriate treatment. The ability to quickly and accurately identify drug-resistant strains could improve patient outcomes by enabling timely and targeted antifungal therapy. Thus, despite its limitations, this study represents an important step towards improving the diagnosis and treatment of fungal infections. Further research with larger and more balanced sample sizes is needed to validate these findings and to fully realize the potential of spectroscopic markers in clinical diagnostics.

## Materials and methods

The *Candida* strains came from the Laboratory of the Chair and Department of Medical Microbiology, Medical University of Lublin. The fungi were stored at -70 °C in VIABANK cryovials (BioMaxima).

### Susceptibility testing

The minimum inhibitory concentration (MIC) of the tested drugs was determined using The Liofilchem^®^ MTS™ (MIC Test Strip) - antifungal concentration gradient strips^75^ on the RPMI1640 medium supplemented with 2% glucose (bioMerieux) using a 0.5 McFarland inoculum suspension in physiological saline. The MIC values were read after 24 h (confirmed after 48 h) of incubation in aerobic conditions at 35 °C. Antifungal susceptibility tests were performed in duplicate. MIC reading was carried out in accordance with the test strip manufacturer’s recommendations: Amphotericin B – the MIC value was read at the complete growth inhibition; Azoles – at the first point of significant inhibition/significant decrease in growth density (80% inhibition); Echinocandins: at the point of significantinhibition (i.e. 80%). The reference strain *Candida parapsilosis* ATCC 22,019 was used for the control and MIC values were interpreted in accordance with the EUCAST recommendations.

### ATR-FTIR analysis

*Candida* strains were grown on Sabouraud Agar with chloramphenicol at 37 °C for 24 h. *Candida* colonies were taken from 1 petri dish and fixed in 2.5% paraformaldehyde (PFA) overnight. The optical density of the suspensions was adjusted to 0.9. Before measurement, 200 µl of each suspension was taken and centrifuged at 5700 rpm for five minutes. The PFA was removed and the remaining inoculum was washed three times with 300 µl of PBS and twice with 300 µl of deionized water, with centrifugation at 5700 rpm for five minutes after each wash.

The amount of 100 µl of the final suspension from each sample was dispensed onto a ZnSe crystal and air-dried. ATR-FTIR spectra spanning the wave number range of 4000–900 cm^− 1^ were collected for all analysed samples using the FTIR VERTEX 70 spectrometer (Bruker Optic GmbH, Ettlingen, Germany) equipped with an MCT detector. Each spectrum was acquired with a spectral resolution of 2 cm^− 1^ and an average of 64 scans were recorded for each sample and background spectrum. Each isolate sample was measured three times.

All ATR-FTIR spectra were baseline-corrected using the concave rubberband correction method in OPUS 7.5 software (Bruker Optic GmbH, Ettlingen, Germany), with 10 iterations and 64 baseline points. Smoothing was performed using the Savitzky-Golay filter (13 smoothing points), followed by vector normalization and offset correction. Reversed second derivatives were calculated using the same smoothing parameters and subsequently vector-normalized.

Determination of band intensities in reversed second derivatives were carried out using OPUS 7.5 software (Bruker Optic GmbH, Ettlingen, Germany). For each identified band, the integration interval was defined based on the extreme positions of the peak in the mean spectrum of each *Candida* species. These intervals were applied consistently across all isolates within the species.

The graphical processing of spectra and their reverse second derivatives was performed using the Origin Pro 2020b program (OriginLab Corporation, Northampton, MA, USA).

### Statistical analysis

The statistical analysis of changes in the IR spectra of *Candida albicans*, *Candida glabrata* and *Candida dubliniensis* isolates, comparing antifungal-resistant strains to sensitive ones, was conducted using STATISTICA 7.1 software (StatSoft, Inc., 2005, Tulsa, OK, USA). The non-parametric Mann–Whitney U test was employed to evaluate the statistical significance of differences in the biomolecular content between resistant and sensitive isolates. The results were analysed at significance levels of *p* < 0.05 and *p* < 0.1. The non-parametric test was chosen because the data did not meet the assumptions of normality and equal group sizes required for parametric testing. The use of *p* < 0.1 as an additional significance level was justified in this exploratory phase of the research to detect potential trends and directions for further investigation, especially given the limited sample size and the preliminary nature of the study. This approach helps to identify promising markers that warrant more extensive studies with larger sample sizes in the future.

## Data Availability

The data can be available upon request from the corresponding author.
